# Supplementary material for: Multimodal instruction with AI-generated images for noun retention: Exploring semantic scene and materiality effects
Source: PLoS One. 2026 Apr 2;21(4):e0334778. doi: 10.1371/journal.pone.0334778 (PMC13046138; doi:10.1371/journal.pone.0334778)
Supplement: Attachment — Submitted filename: Response to Reviewers.docx [file pone.0334778.s002.docx]

PONE-D-25-52191

Response to Reviewers

Multimodal Instruction with AI-Generated Images for Noun Retention: Exploring Semantic Scene and Materiality Effects

We thank the Academic Editor Dr. Ramandeep Kaur and Reviewer Mr. Jithin Balan for your constructive and detailed comments. We have addressed every point below; all changes are highlighted in the revised manuscript with Track Changes.

**1. Abstract: “AIGC” not expanded at first mention**

RESPONSE: Expanded at first occurrence as “artificial intelligence (AI)-generated visual content” (lines 15).

**2. Typographical error “approximately” on p. 2**

RESPONSE: Corrected to “approximately” (line 40).

**3. Last paragraph on p. 4 (old ms) outlines paper structure**

RESPONSE: Rewritten to provide rationale and objectives only; section preview removed (lines 106–113).

**4. Missing reference for “traditional multimodal materials” (p. 5)**

RESPONSE: Added Mayer, R. E. (2009) as foundational citations (lines 133,540-541).

**5. Introduction lacks balanced review of AI-image limitations**

RESPONSE: Added new subsection “Potential Limitations of AI-Generated Visuals” (lines 75–92) citing:

“Indeed, the educational promise of these multimodal tools is accompanied by well-documented technical limitations.

Perceptual inconsistency: AI models frequently produce distorted objects or illogical spatial relations that may increase extraneous cognitive load [11].

Socio-cultural bias: generated images tend to over-represent specific cultural archetypes, reinforcing stereotypes rather than fostering cross-cultural understanding [12].

Output variability: fluctuations in colour fidelity and detail introduce unintended perceptual noise, while the detectability of synthetic images changes over their online lifespan, complicating responsible use [13].

All references are already present in the provided reference list.

**6. Methodological flaw: only unimodal vs multimodal**

RESPONSE:

- Design clarified: two-group (text-only vs. text + AI-images) (lines 184–185).

Conclusions rewritten throughout to attribute effect to “multimodal presentation” rather than “AI-generated nature” (see Abstract, Discussion, Conclusion).

**7. Participant demographics missing**

RESPONSE: Participants subsection (lines 166–179) report:

N = 40 university students; native Chinese speakers; ≥ 6 years formal English.

All participants majored in either big data & accounting (n = 20) or engineering cost management (n = 20).

No colour-vision or visual impairments

Ethics approval No. CJ-202412003 from the College of Urban Construction,Anhui Vocational College of Defense Technology ; written informed consent obtained.

**8. Misleading conclusion about “AIGC-enhanced” superiority**

RESPONSE:We fully accept the reviewer's critique. The original manuscript did indeed obscure the independent variable under investigation. Our experimental design was specifically a comparison between two modal conditions: "text-only" and "text-plus-image," not a comparison of image sources.

The major revisions we have implemented include:

Conceptual Clarification: Throughout the manuscript, particularly in the Introduction and Discussion, we have clarified that this study aims to investigate the effectiveness of "a multimodal pedagogical approach implemented using AIGC tools," rather than testing AIGC-generated imagery in isolation.

Terminology Correction: We have systematically revised terms such as "AIGC-enhanced instruction/approach/group" to more accurate descriptors like "the multimodal condition/instruction/group (employing AI-generated images)" or "the text-with-image condition."

Conclusion Rewriting: We have thoroughly rewritten the Conclusion. The key finding is now stated as: "Compared with traditional text-based instruction, the multimodal presentation led to better recall..." This explicitly attributes the advantage to the multimodal presentation itself, not to AIGC per se. (Please see the revised conclusion section.)

Title Adjustment: To maximize precision, we propose a slight adjustment to the main title: "Multimodal Instruction with AI-Generated Images for Noun Retention: Exploring Semantic Scene and Materiality Effects.".

**9. Image-to-word matching task inappropriate for text-only group**

RESPONSE: We agree with the reviewer's concern and have revised the manuscript to address it. The “image-to-word matching” task is now explicitly defined as a cross-modal transfer task designed to assess the quality of learners' mental representations, rather than a direct test of trained skills. This clarification appears in the Methods (lines 272-278). Accordingly, the large performance difference (0.85 vs. 0.45) is interpreted not as a simple advantage, but as evidence that the multimodal condition supported the formation of more robust and flexibly accessible semantic representations (see Results, lines 375-383).

--------------------------------------------------------------------

ADDITIONAL EDITOR / JOURNAL REQUIREMENTS

 File naming & style – checked with PLOS template; all filenames now conform.

 Participant consent – added explicit statement: “Written informed consent was obtained; consent form filed in participant folder” (lines 174–176).

Code availability – All analyses were conducted in Microsoft Excel; the annotated calculation sheets and formulas are publicly available at Protocols.io (DOI: dx.doi.org/10.17504/protocols.io.j8nlk1bx6g5r/v2).

Data availability – This study shares research materials to the extent permitted:

Full Methodology: The complete, step-by-step experimental protocol is available on Protocols.io (DOI: dx.doi.org/10.17504/protocols.io.j8nlk1bx6g5r/v2).

Analysis Materials: The Excel workbook (experiment_results.xlsx) containing raw data and all calculations is deposited in the same Protocols.io project.

Research Data: The original, de-identified trial-level data are stored on a secure institutional platform at [the College of Urban Construction，Anhui Vocational College of Defense Technology]. Due to current platform policies and data governance agreements, these raw data cannot be exported or deposited in a public repository. As a complete alternative, all data necessary to replicate the reported statistical findings—including the values behind all means, standard deviations, and test statistics—are provided in the supporting information files experiment_results.xlsx (located in 5. Supplemental Materials). For further inquiries regarding the original raw data, qualified researchers may contact the corresponding author or the designated data access office of the College of Urban Construction at [cjxsgz@acdt.edu.cn]. The institutional ethics committee at [35311296@acdt.edu.cn] may also be contacted for matters pertaining to research ethics and compliance oversight related to data sharing.

 Figure 1 participant image – In full compliance with PLOS policies on publication consent, the original Figure 1 has been replaced with a different image, as obtaining informed consent for the publication of an AI-generated likeness was not feasible.

 Typographical & reference errors – Full spell-check performed; “approximately” and missing ref. fixed (see #2, #4 above).

--------------------------------------------------------------------

We appreciate the opportunity to improve our manuscript and believe the revised version fully addresses the methodological, reporting, and ethical concerns raised.

Sincerely,

Corresponding Author: Yan Shibo

On behalf of all co-authors
